# Evolution of morphological crypsis in the *Tetramorium caespitum* ant species complex (Hymenoptera: Formicidae)

**DOI:** 10.1038/s41598-018-30890-z

**Published:** 2018-08-22

**Authors:** Herbert C. Wagner, Alexander Gamisch, Wolfgang Arthofer, Karl Moder, Florian M. Steiner, Birgit C. Schlick-Steiner

**Affiliations:** 1 Department of Ecology, University of Innsbruck, Technikerstraße 25, 6020 Innsbruck, Austria; 2 Department of Biosciences, University of Salzburg, Hellbrunnerstraße 34, 5020 Salzburg, Austria; 3 Institute for Applied Statistics and Computing, Department of Landscape, Spatial and Infrastructure Sciences, Boku, University of Natural Resources and Life Sciences Vienna, Peter-Jordan-Straße 82/I, 1190 Vienna, Austria

## Abstract

Cryptic species are morphologically very similar to each other. To what extent stasis or convergence causes crypsis and whether ecology influences the evolution of crypsis has remained unclear. The *Tetramorium caespitum* complex is one of the most intricate examples of cryptic species in ants. Here, we test three hypotheses concerning the evolution of its crypsis: H1: The complex is monophyletic. H2: Morphology resulted from evolutionary stasis. H3: Ecology and morphology evolved concertedly. We confirmed (H1) monophyly of the complex; (H2) a positive relation between morphological and phylogenetic distances, which indicates a very slow loss of similarity over time and thus stasis; and (H3) a positive relation between only one morphological character and a proxy of the ecological niche, which indicates concerted evolution of these two characters, as well as a negative relation between p-values of correct species identification and altitude, which suggests that species occurring in higher altitudes are more cryptic. Our data suggest that species-specific morphological adaptations to the ecological niche are exceptions in the complex, and we consider the worker morphology in this complex as an adaptive solution for various environments.

## Introduction

Morphological crypsis was detected in birds 300 years ago^1^ but considered as a marginal phenomenon until the late 20^th^ century^2^. Due to morphometric^3–6^ and molecular-genetic^7,8^ improvements in the last decades, morphological crypsis is now known as widespread not only in animals but in all clades of life^2,9^. At least for the human observer, cryptic species look very similar to each other so that safe determination based on qualitative morphology is impossible^2,5,10^. Cryptic species are taxonomically non-randomly distributed^2,9^, and in some animal taxa, they might represent about the half of all species^5,10,11^. Speciation rates and ages of species are considered to be important factors affecting crypsis, but morphological crypsis can mask species boundaries even if evolutionary distances are large^2,12^. Only after these boundaries have been decrypted reliably, evolutionary patterns that create crypsis can be revealed^13^. Little is known about the frequency of the two most important routes into morphological crypsis, stasis, that is, retention of ancestral morphology despite genetic differentiation, and convergence, that is, acquisition of a similar morphological trait in different lineages^2,9,13^. The strength of ecologically mediated selection pressure on morphology often remains unclear^14–17^. Possible routes of morphological evolution are interspecific congruences as adaptation to ecological niches^17–19^ but also character displacement, that is, a divergence of traits mediated by ecological similarities^20,21^.

**Figure 1:**
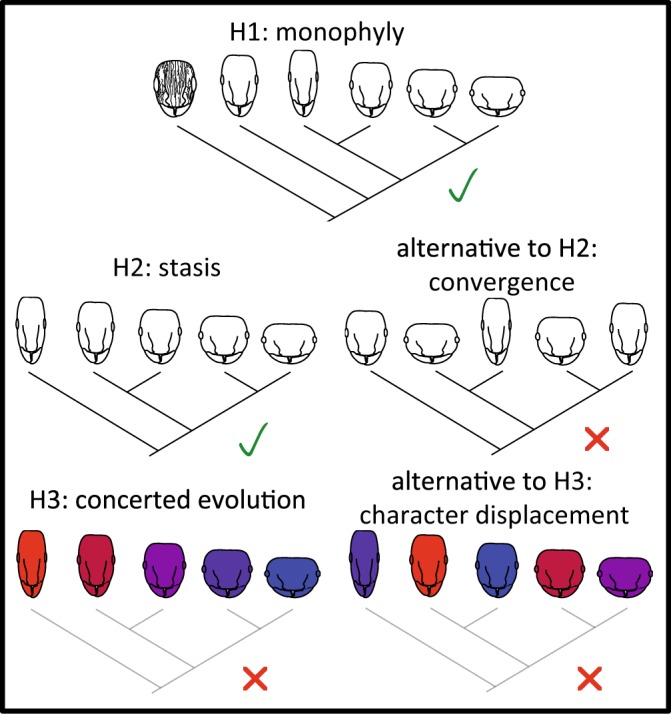
Hypotheses H1–H3. H1: The species complex is monophyletic (confirmed). H2: Morphology resulted from evolutionary stasis (confirmed). Alternative to H2: Morphology evolved convergently (rejected). H3: Ecology and morphology evolved concertedly (rejected). Alternative to H3: The evolution of ecology and morphology was mediated by character displacement (rejected; for details, see Results and Discussion). Head with rugae and larger eyes symbolizes species outside the *T*. *caespitum* complex. Head shapes symbolize morphology within the *T*. *caespitum* complex, colours from blue to red ecology. The grey colour of the species tree in H3 shows this factor was corrected for in a Partial Mantel test (see Methods, Statistics).

The family of ants contains many cryptic species^5,22^. The genus *Tetramorium* comprises more than 500 species worldwide^23^. The *Tetramorium caespitum* group sensu Bolton (1995) includes at least four cryptic species complexes in the Palearctic^24–28^. One of these, the *Tetramorium caespitum* complex, is one of the most intricate examples of cryptic ant species complexes^22,24,25,29–32^, comprising at least eleven species^25,30,33^. All these species are cryptic since always at least one other species of the complex comes into question for a misidentification, and no safe identification is possible without quantitative morphometric analysis^22,25,30^. However, throughout Europe, the species do consistently differ in mitochondrial DNA, nuclear DNA, selected characters of worker morphology, male genitals, and ecology^25^. Thus, they fulfill criteria of species under the unified species concept^34^. Even between species of this complex and species outside the complex, a safe morphological differentiation is not always simple^25,27^ and can require several worker characters, gyne size, and male genital morphology^25^. Recently, a large-scale set of morphometric, nuclear genetic, and distribution data became available^25^, making the *T*. *caespitum* complex well suited for studying the evolution of morphological crypsis. In the following, we evaluate three hypotheses (H1–H3) concerning the evolution of crypsis in this complex (Fig. 1).

## Hypotheses about the evolution of the ***Tetramorium caespitum*** complex

### Hypothesis H1: The complex is monophyletic

While the *T*. *caespitum* complex can be delimited morphologically from other species of the *Tetramorium caespitum* group^25,27^, it is, according to mitochondrial DNA (mtDNA) phylogenies, not monophyletic with respect to other species of the group^24,29,32^. Using the same set of species as for those mtDNA phylogenies, we reconstruct a SNAPP^35^ phylogeny based on nuclear data to decide if the nuclear-DNA (nuDNA) based tree is in line with the morphological species-complex definition and thus mtDNA is unsuitable to resolve the species complex’ topology with respect to other species of the *T*. *caespitum* group (cf. Wagner *et al*.^25^).

### Hypothesis H2: Morphology resulted from evolutionary stasis

A positive relation between morphological and phylogenetic distances within the complex would state that morphological similarity is lost over evolutionary time, even if very slowly and even though the species are currently very similar. It would thus indicate stasis. In contrast to stasis, a negative relation between morphological and phylogenetic distances, that is, less morphological similarity between genetically closer related species and vice versa, would indicate convergence of morphological traits^13,17^. Stasis (H2) and its alternative hypothesis, convergence, are mutually exclusive. A lack of significance might either indicate a positive correlation in some and negative correlation in other species or an entire lack of correlation. To test H2, possible relations are assessed with Mantel tests.

### Hypothesis H3: Ecology and morphology evolved concertedly

A positive relation between morphological and ecological distances would suggest concerted evolution, that is, correlation of distances in one or more species between multiple character suites caused by evolution^18,36^. In more detail, morphological traits would have an adaptive value in a certain environment or ecological-niche traits would have an adaptive value given certain morphological character traits. In contrast to concerted evolution, a negative relation between morphological and ecological distances might indicate character displacement^20,21^, which describes the avoidance of competition by utilizing different micro-ecological niches in sympatry, eventually leading to evolution of differences in morphology even if minor. Concerted evolution (H3) and its alternative hypothesis, character displacement, are mutually exclusive. A lack of significance might either indicate a positive correlation in some and negative correlation in other species or an entire lack of correlation. To test H3, possible relations between morphological distance and several ecological parameters, that is, climatic variables, soil variables, altitude, and latitude, are investigated in this study by performing linear regressions and Partial Mantel tests.

## Methods

### Study organisms and origin of data

Workers of eleven cryptic ant species of the *Tetramorium caespitum* complex were used as study system (Table 1)^25,33^. Material from 1385 localities from 43 countries was included. For morphometrics, the present study capitalizes on 29 continuous and two discrete characters of 990 individuals. These individuals belong to 464 nests of ten species (no data for the east Palaearctic *T*. *tsushimae*) from a previous study^25^. For phylogenetics, 890 amplified fragment-length polymorphism (AFLP) loci of 269 individuals belonging to ten species (no data for *T*. *breviscapus*) were extracted from the same source^25^. Compatible, hitherto unpublished AFLP data of five species outside the *T*. *caespitum* complex (Table 1) yielded in the same AFLP runs were merged with that dataset. As material of some species is difficult to acquire, we used one representative species of every defined species complex of the *T*. *caespitum* group. The rationale in using just one representative species per complex (except in the *T*. *caespitum* complex) was that, in the case that indeed members of one of the well-delimited complexes (e.g., *T*. *chefketi* complex^26^) would fall into the *T*. *caespitum* complex, it would rather be parsimonious that the whole well-delimited complex and not just a single species of it would fall into the *T*. *caespitum* complex. Ecological data were newly established using 20 climatic variables, eight soil variables, and 1385 published sampling localities^25^ from ten species (no data for *T*. *tsushimae*) and using only one sample per species and locality (=1106 species-locality combinations; Table 2). The datasets generated and analysed during the current study are available from the corresponding author on request.

**Table 1:** Species used in this study with information on taxon affiliation (complex or group)^25–28,87^, distribution^25–28,33,87–89^, altitude^25^, and sample size for morphometrics, phylogenetics, and ecology.

Species	Higher-level taxon	Distribution	Altitude [meter above sea level]: arithmetic means [lower extreme, upper extreme]	Sample size [nests]: morphometrics; phylogenetics; ecology
*Tetramorium alpestre* Steiner *et al*., 2010	*Tetramorium caespitum* complex sensu Wagner *et al*.^25^	Iberia, France, Central Europe, Italy, Balkans	1856 [970–2400]	73; 44; 174
*Tetramorium caespitum* (Linnaeus, 1758)		Europe and Caucasus	586 [1–2100]	145; 79; 445
*Tetramorium hungaricum* Röszler, 1935		Central Europe, Balkans, Eastern Europe	328 [27–940]	23; 12; 28
*Tetramorium breviscapus* Wagner *et al*., 2017		Balkans	210 [3–527]	3; 0; 3
*Tetramorium indocile* Santschi, 1927		Iberia, France, Central Europe, Italy, Balkans, Eastern Europe, Caucasus, Central Asia	1351 [110–2300]	43; 22; 38
*Tetramorium caucasicum* Wagner *et al*., 2017		Caucasus	2009 [1275–2500]	10; 6; 7
*Tetramorium fusciclava* Consani & Zangheri, 1952		Italy	101 [1–1200]	17; 8; 12
*Tetramorium staerckei* Kratochvíl, 1944		Central Europe, Balkans, Eastern Europe, Central Asia	623 [1–2320]	41; 31; 73
*Tetramorium impurum* (Foerster, 1850)		Iberia, France, Central Europe, Benelux, Italy, Balkans, Anatolia	909 [1–2235]	78; 39; 148
*Tetramorium immigrans* Santschi, 1927		Iberia, France, Central Europe, Italy, Balkans, Eastern Europe, Anatolia, Caucasus, both Americas	285 [0–2100]	40; 28; 178
*Tetramorium tsushimae* Emery, 1925		Eastern Asia, North America		0; 2; 0
*Tetramorium ferox* Ruzsky, 1903	*Tetramorium ferox* complex sensu Csősz & Schulz, 2010	Central Europe, Balkans, Eastern Europe, Caucasus, Anatolia		0; 2; 0
*Tetramorium moravicum* Kratochvíl, 1941	*Tetramorium chefketi* complex sensu Csősz *et al*., 2007	Balkans, Eastern Europe, Anatolia, Caucasus		0; 2; 0
*Tetramorium semilaeve* André, 1883	*Tetramorium semilaeve* complex sensu Csősz & Schulz, 2010	Iberia, France, Italy		0; 2; 0
*Tetramorium bicarinatum* (Nylander, 1846)	*Tetramorium bicarinatum*-group sensu Bolton, 1980	Worldwide (tramp species)		0; 2; 0
*Tetramorium caldarium* (Roger, 1857),	*Tetramorium simillimum*-group sensu Bolton, 1980	Worldwide (tramp species)		0; 2; 0

**Table 2:** Quantitative ecological variables used for calculation of intra- and interspecific Euclidian distances.

Variable	Type	Definition
Bio1	Climatic	Annual mean temperature
Bio2		Mean diurnal range (mean of monthly (max temp-min temp))
Bio3		Isothermality (BIO2/BIO7) (*100)
Bio4		Temperature seasonality (standard deviation *100)
Bio5		Max temperature of warmest month
Bio6		Min temperature of coldest month
Bio7		Temperature annual range (BIO5-BIO6)
Bio8		Mean temperature of wettest quarter
Bio9		Mean temperature of driest quarter
Bio10		Mean temperature of warmest quarter
Bio11		Mean temperature of coldest quarter
Bio12		Annual precipitation
Bio13		Precipitation of wettest month
Bio14		Precipitation of driest month
Bio15		Precipitation seasonality (coefficient of variation)
Bio16		Precipitation of wettest quarter
Bio17		Precipitation of driest quarter
Bio18		Precipitation of warmest quarter
Bio19		Precipitation of coldest quarter
TAS		Thermal niche sensu Seifert & Pannier (2007)^50^
Bldfie	Soil	Bulk density in kg/m^3^
Cecsol		Cation exchange capacity in cmolc/kg
Clyppt		Clay content mass fraction
Crfvol		Coarse fragments volumetric
Ocstha		Soil organic carbon stock in t/ha
Phihox		Soil pH value in 10x in H_2_O
Sltppt		Silt content mass fraction
Sndppt		Sand content mass fraction

### Phylogenetic analyses

Species trees were reconstructed using two different approaches. AFLP profiles were computed with SNAPP v1.3.0^35^. SNAPP is a multispecies coalescent framework that uses genetic markers (e.g., biallelic SNPs or AFLP banding patterns) to estimate species trees with (relative) divergence times and population sizes^35^. *Tetramorium bicarinatum* and *T*. *caldarium* (Table 1) were used as outgroups. Due to the computational demands of the algorithm^35^, a representative subsample of the AFLP dataset (32 workers; two workers per species) was selected. Model parameters for instantaneous rates of forward (from allele 1 to 0) and backward (from allele 0 to 1) mutations were calculated based on the data matrix while the remaining parameters were left at their default values. Six independent Markov chain Monte Carlo (MCMC) chains, each with 500,000 generations and sampling every 100 generations, were run using the SNAPP package as implemented in BEAST v2.4.3^37^ and combined using Log Combiner v2.4.3 after the removal of 10% burn-in^38^. The MCMC samples were inspected in Tracer v1.6^39^ to investigate convergence of the chain to stationarity and assess sampling adequacy. The results of the SNAPP analyses were summarized as a maximum clade credibility tree with median node heights using Tree Annotator v2.4.3^40^ and visualized in FigTree v1.4.2^41^. The posterior distributions of the SNAPP consensus species trees were visualized using DensiTree v2.2.0^42^. Only nodes supported by Bayesian posterior probabilities ≥0.95 were accepted as monophyletic. Monophyly of the complex (H1) is not formally required for testing H2 and H3.

Additionally, seven nuclear genes (Supplementary Table S1), previously used in the classification of myrmicine ants^43^, were sequenced. Sequencing was performed for all *Tetramorium* species included in this study except *Tetramorium semilaeve*, for which PCR failed in several of these genes. The primers used for amplifying and sequencing were the same as in Ward *et al*.^43^; the PCR settings followed Ward *et al*.^43^ with modifications of the annealing temperatures (52 °C for Wingless and abdominal A; 55 °C for the other genes). After adding *Tetramorium spinosum* (Pergande, 1896) as additional outgroup (GenBank accession numbers KJ859859, KJ860664, KJ861127, KJ861322, KJ861506, KJ861935, KJ861737), all sequences were aligned with Clustal W2^44^ using default settings and concatenated manually. Partitions were set according to genes and codon positions, and model selection using the edge-unlinked partition mode (each partition has its own set of branch lengths) was performed automatically before Maximum Likelihood tree construction using the web interface of IQ-TREE^45^. For assessing node support, ultrafast bootstrapping and Shimodaira-Hasegawa branch length tests with 1000 iterations were applied (Supplementary Figure S1).

### Statistics

For calculating morphological distance, 31 morphological characters were divided by the head-index CS (character abbreviations defined in Supplementary Table S2) and used to build arithmetic nest means, which were further analysed in two approaches: (i) For an all-character morphological distance, characters were reduced by a principal component analysis in PAST v3.06^46^. Principal components that explained cumulatively at least 80% of variance were used to calculate Euclidian distances among all nests in SPSS Statistics v21 (IBM, USA). (ii) For single-character morphological distances, all morphological characters were used individually to calculate Euclidian distances. Two character pairs correlated, CW/CS with CL/CS (R < −0.8) and SPST/CS with MPSP/CS (R > 0.8); from each pair, only one randomly selected character (i.e., CL/CS and MPSP/CS) was retained. Self-comparisons (distance ‘0’) were excluded from the matrices of approaches (i) and (ii), and arithmetic means of intra- and interspecific Euclidian distances were calculated. For phylogenetic distance, the binary AFLP matrix of 269 workers was reduced by a principal component analysis. Principal components that explained cumulatively 80% of variance were used to calculate Euclidian distances among all nests. Self-comparisons were excluded from the matrices, and arithmetic means of intra- and interspecific Euclidian distances were calculated.

Ecological distances of samples were based on 20 climatic variables for current conditions (~1960–1990) and eight soil variables^47,48^. The climatic variables consisted of the 19 bioclimatic variables Bio1–19 from the WorldClim database v1.4^49^ at 30 arc-seconds resolution and the standard air temperature (TAS^50^, Table 2) from a previous study^25^. TAS is the mean air temperature at two meters height observed from 1 May to 31 August; 1961–1990 data were taken from proximate meteorological stations and corrected for longitude, latitude, and altitude^50^. The ecological variables were extracted for each of the 1106 sampling localities using the R-package Raster v2.5–8^51^. Euclidian distances were calculated as described above for the all-character morphological distance.

Possible correlations were tested by Mantel tests between all-character and single-character morphological distances vs. phylogenetic distances in PAST using 100,000 permutations. A potential correlation between morphological and ecological distances was tested in a Partial Mantel test correcting for phylogenetic distances.

Arithmetic nest means (464 nests) of morphometric data (31 characters) were used to calculate p-values of correct species identification in a discriminant analysis in SPSS. Two linear regressions were calculated in SPSS, using as independent variables (i) altitudes and (ii) latitudes from a previous study^25^ and as dependent variable the p-values for the correct species identification from the discriminant analysis.

An α-level of 0.05 was used; in cases of multiple comparisons with single-character morphological distances, Bonferroni-Holm correction^52^ was applied. Bonferroni-Holm correction is employed to control the family-wise type-I-error rate but it increases the rate of Type-II errors^52,53^. Therefore, also the probability $$prob=\sum _{i=k}^{n}$$$$(\begin{array}{c}n\\ i\end{array}){p}^{i}{(1-p)}^{n-i}$$ to receive by chance the number of single characters with p ≤ 0.05 as seen in this study was calculated (cf. Bernoulli^54^, cf. Moran^53^). In this equation, *n* is the total number of characters (29), *p* is 0.05, and *k* the number of characters with p ≤ 0.05 (11, 3; see Results).

## Results

### Hypothesis H1: The complex is monophyletic

**Figure 2:**
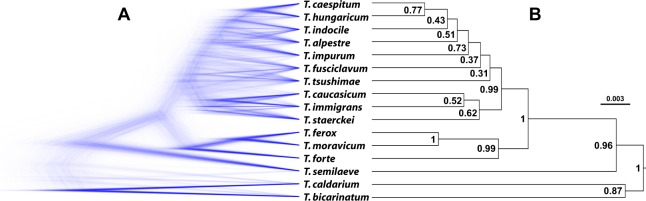
SNAPP phylogeny. Analyses of species trees of AFLP data in the multispecies coalescent framework as implemented in SNAPP: **(A)** complete set of consensus trees; **(B)** maximum clade credibility tree. Posterior probabilities are given at nodes.

Evaluation of the combined SNAPP results revealed an effective sample size (ESS) larger than 200 for most of the parameters estimated by the MCMC chains, indicating convergence. The only two exceptions, ‘height of the tree’ and ‘ancestral population size’ for the node of the clade *Tetramorium forte*/*T*. *caespitum*, were likely inconsequential for this analysis as they affect parameters other than topology and posterior probabilities (PPs) (personal communication, A. Rambaut) of species belonging to the *T*. *caespitum* complex. The multispecies coalescent species tree revealed some supported relationships between lineages, especially at the basal nodes of the phylogeny (Fig. 2). The *T*. *caespitum* complex was retrieved as monophylum (PP = 0.99, 10 species) and was reconstructed to have a common ancestor also using sequences of seven nuclear genes previously used in the classification of myrmicine ants^43^ (Supplementary Figure S1, Supplementary Table S1). The intracomplex relationships, however, remained unresolved (PPs ranging from 0.31 to 0.77, Fig. 2), and this did not change when using the seven nuclear genes (Supplementary Figure S1).

### Hypothesis H2: Morphology resulted from evolutionary stasis

We found a positive correlation between all-character morphological and phylogenetic distances (Mantel test, R = 0.589, p = 0.035, 9 species, Table 3). In 29 single-character comparisons, no significant correlation remained after a Bonferroni-Holm correction (Mantel test, p > 0.005 in all characters, 9 species, Table 3). Eleven single characters correlated positively with phylogenetic distance at p < 0.05; the probability to see at least eleven out of 29 characters at p ≤ 0.05 by chance (Mantel test, 9 species, Table 3) was very low (*prob* < 10^−8^). These characters had been taken from head (CL, EL, EW, PreOc; character abbreviations defined in Supplementary Table S2), mesosoma (MW, MtpW, MPSP, MPPL), petiole (PEW, PEL), and gaster (MC1TG).

**Table 3:** Results of Mantel tests between morphology and phylogenetics and Partial Mantel tests (corrected for phylogenetics) between morphology and a proxy of the ecological niche.

Morphological characters	Phylogenetics (Mantel tests)	Ecology (Partial Mantel tests)
All-character morphology	**R = 0**.**589**, **p = 0**.035*	R = 0.186, p = 0.232
HFL/CS	R = 0.204, p = 0.206	R = −0.077, p = 0.504
ML/CS	R = 0.338, p = 0.146	R = −0.093, p = 0.522
PPW/CS	R = 0.311, p = 0.146	R = 0.146, p = 0.192
PEW/CS	**R = 0**.**574**, **p = 0**.**027**	R = 0.361, p = 0.090
SPWI/CS	R = 0.569, p = 0.064	**R = 0**.**653**, **p = 0**.**021**
MtpW/CS	**R = 0**.**486**, **p = 0**.**020**	R = 0.216, p = 0.149
MW/CS	**R = 0**.**652**, **p = 0**.**010**	R = 0.216, p = 0.154
CL/CS	**R = 0**.**543**, **p = 0**.**037**	R = −0.064, p = 0.524
PoOc/CS	R = 0.551, p = 0.063	R = 0.12, p = 0.282
FL/CS	R = 0.33, p = 0.158	R = −0.285, p = 0.906
dAN/CS	R = 0.123, p = 0.359	R = −0.307, p = 0.942
RTI/CS	R = 0.627, p = 0.055	R = 0.179, p = 0.214
SLd/CS	R = 0.331, p = 0.145	R = −0.091, p = 0.470
POTCos/CS	R = 0.252, p = 0.192	R = −0.135, p = 0.644
EW/CS	**R = 0**.**461**, **p = 0**.**037**	R = 0.334, p = 0.109
EL/CS	**R = 0**.**658**, **p = 0**.**012**	R = 0.284, p = 0.162
PreOc/CS	**R = 0**.**565**, **p = 0**.**007**	R = 0.175, p = 0.167
PEH/CS	R = 0.136, p = 0.296	R = 0.272, p = 0.141
PEL/CS	**R = 0**.**49**, **p = 0**.**041**	R = 0.141, p = 0.202
PPL/CS	R = 0.205, p = 0.220	R = −0.123, p = 0.621
PPH/CS	R = 0.161, p = 0.277	R = 0.026, p = 0.364
PnHL/CS	R = 0.232, p = 0.147	R = 0.004, p = 0.416
Ppsh/CS	R = 0.159, p = 0.337	R = −0,114, p = 0.534
MPSP/CS	**R = 0**.**611**, **p = 0**.**024**	**R = 0**.**46**, **p = 0**.**046**
MPST/CS	R = 0.202, p = 0.257	R = −0.066, p = 0.393
MPPL/CS	**R = 0**.**584**, **p = 0**.**012**	R = −0.016, p = 0.442
PLST/CS	R = 0.37, p = 0.121	R = −0.274, p = 0.859
PLSP/CS	R = 0.187, p = 0.219	**R = 0**.**664**, **p = 0**.001*
MC1TG/CS	**R = 0**.**558**, **p = 0**.**028**	R = 0.183, p = 0.237
**Probability**	***prob* < 0**.**00000001**	*prob* = 0.175

Characters with p ≤ 0.05 in bold, significances after Bonferroni-Holm correction with *. Probability (*prob*) to receive by chance the number of single characters with p ≤ 0.05 as seen in this study is given.

### Hypothesis H3: Ecology and morphology evolved concertedly

A Partial Mantel test yielded no significant correlation between all-character morphological and ecological distances (R = 0.186, p = 0.232, 10 species, Table 3). One single morphological character, that is, the distance between the most dorsocaudal point of propodeal lobe and the distalmost point of propodeal spine (PLSP), correlated positively with the ecological niche after Bonferroni-Holm correction (R = 0.664, p = 0.001, 10 species, Table 3). Three characters influenced by propodeal-spine shape (MPSP, PLSP, SPWI; Table 3) had p ≤ 0.05; the probability for at least three out of 29 characters to be at p ≤ 0.05 by chance (Mantel test, 10 species, Table 3) was high (*prob* = 0.175). Linear regressions of p-values dependent on the absolute values of altitudes and of latitudes yielded a negative (R = −0.3054, p < 10^−11^, 464 nests) and a positive result (R = 0.1105, p = 0.0124), respectively.

## Discussion

### Hypothesis H1: The complex is monophyletic

The monophyly of the *Tetramorium caespitum* complex with respect to the selected set of *T*.*-caespitum*-group species outside the complex is supported by nuDNA and the morphology of workers, gynes, and males^25^ but contradicted by published mtDNA phylogenies^24,29,32^. The mtDNA polyphyly of some species of the *T*. *caespitum* complex was already explained by peripatric speciation or historical introgression^25^. The same might hold true also for species outside of the complex. We cannot rule out other reasons for mtDNA polyphyly, including signal saturation^55^ and long-branch attraction^56^; however, both seem unlikely due to a maximum intercomplex mtDNA-divergence of just 10.6%^24^. Although the relationship of the *T*. *caespitum* complex with other species supports its monophyly with respect to the selected species outside the complex, AFLP data are insufficient to reconstruct any topology within the complex, so that not a single sister relationship within the complex is known. Possibly, ongoing interspecific hybridization^25^ affects genotypes and thus weakens intracomplex node support. Anyway, for the evaluation of H1, the intracomplex relationships are inconsequential.

Since we included members of the most relevant species complexes in this study, the monophyly might hold true with respect to all *Tetramorium* species. However, the social parasites using as hosts species of the *Tetramorium caespitum* complex must be treated with care here. These species are non-cryptic^22^ but phylogenetically related to the complex^43,57^. Future studies focusing on the phylogeny of these parasites may uncover their true relation to the species of the *T*. *caespitum* complex. Whatever the future outcome will be, it will not be relevant to solving our research question, given the completely different selection pressure acting on social parasites and their highly derived morphology.

Monophyly is in line with the hypothesis that crypsis evolved by stasis (H2). In contrast, a polyphyletic relationship of cryptic species would have necessitated morphological convergence as explanation.

### Hypothesis H2: Morphology resulted from evolutionary stasis

The positive correlation between all-character morphological and phylogenetic distances suggests a loss of all-character morphological similarity over evolutionary time. While no single morphological character provided a significant correlation with phylogenetic distance, eleven out of 29 characters had positive correlations with p-values below 0.05, which is unlikely to be a random effect considering the result of the probability test. These characters are distributed over the whole ant body; we consider this as an indicator that current evolutionary change affects not just a part of but the entire body. Interestingly, stabilizing selection can maintain stasis of important morphological characters^2,58^. Based on the morphological crypsis in the *Tetramorium caespitum* complex^24,25^, we speculate that stabilizing selection on worker morphology might play a role and that the morphology of a worker from the complex represents an adaptive solution in a broad habitat spectrum in different environments from southern European coasts up to Alpine mountain habitats^25,32^. Consequently, adaptive phenotypical differences between species to use ecological niches should be searched mainly outside of worker morphology, for example in physiology. However, also the slight loss of morphological similarity from the ancestral state of the species complex should require some characters not influenced by strong stabilizing selection and thus allowed to diverge^58^.

### Hypothesis H3: Ecology and morphology evolved concertedly

The lack of significance between all-character morphological and ecological distances rejects H3. We conclude that neither morphological adaptation to ecological niches nor ecological adaptation to morphological characters constitutes strong selective pressure to evolve changes in all-character morphology or ecology. This finding could result from positive correlation in some and negative correlation in other species or an entire lack of correlation, maybe caused by a balance between concerted evolution and character displacement. Hence, in the case of this *Tetramorium* species complex, morphological similarities should not be seen as a proxy for ecological similarity in terms of climate and soil properties^17,19^. This does not preclude that species-specific morphological adaptations to other ecological factors may exist (cf. Petchey & Gaston^59^). As far as can be assumed from our data, overall morphology and ecological niche of the *T*. *caespitum*-complex species did not evolve in concert. Additionally, concerted evolution of overall morphology and ecological niche seems unlikely due to the large number of morphological structures together contributing to the highly cryptic morphology, and to our knowledge, such concerted evolution has never been observed in animals. In contrast, concerted evolution of single morphological traits, trait complexes, colours, or courtship songs with ecology, which includes also convergence caused by any ecological selection pressure, has been documented in animals^13,17,60–66^. In our data, propodeal spines (one character out of 29) and ecological niche probably evolved in concert, and for this special case, H3 is supported. Thus, the propodeal spines might be under selection pressure caused by a factor linked with the ecological niches of these species. Ant research is far from understanding adaptiveness of species-specific differences in functional morphology^15,16^. While the function of special morphological structures like mandibles in trap-jaw ants^64^, the clypeal excision in *Tapinoma*^16^, and the metapleural gland of a leaf-cutter ant species^67^ are understood, there are no satisfying answers for other well-known structures like the convergently evolved ventral spines in parasitic myrmicine ants^68^ or species-specific hair numbers in *Lasius* and *Formica*^16^. Functional morphology of spinescence in ants was explained by mechanical defense^15,69^ and skeletomuscular adaptation^14^. Both hypotheses seem unlikely to apply to species of the *T*. *caespitum* complex, since the spines are too short to defend the petiole articulation, and the muscles on the dorsal propodeum do not expand into the propodeal spine cavity.

Regressions of p-values of correct species identification dependent on altitudes and latitudes were significantly negative and positive, respectively. However, the relation of the latter is not very strong (R = 0.1105, p = 0.0124). This result may be explained by higher p-values of correct species identification of species occurring in lower altitudes, for example in *T*. *breviscapus*, *T*. *fusciclava*, and *T*. *immigrans*, than in higher altitudes, for example in *T*. *alpestre*, *T*. *caucasicum*, and *T*. *indocile*. The latter might have an intraspecifically more heterogeneous morphology and/or interspecifically more homogenous morphology. Thus, crypsis increases with altitude. In contrast, nests of species in higher latitudes have an intraspecifically slightly more homogeneous morphology and/or interspecifically slightly more heterogeneous morphology than those occurring in lower latitudes. Thus, crypsis decreases with latitude.

In discussing these patterns, we highlight two factors correlating with altitude, terrain ruggedness, and temperature. First, due to the profile of mountains, the habitat of species occurring in higher altitudes is more strongly fragmented than habitats in lower altitudes, that is, terrain ruggedness increases with altitude. In at least some organisms, geographic isolation in mountainous landscapes decreases gene flow and thus increases genetic drift^70–72^ and accelerates local adaptation^73–75^. From this, higher intraspecific variation between populations and potentially also lower p-values of correct species identification could follow. Such slightly higher intraspecific variation has to be seen in relation to other species within the cryptic species complex and thus is not in conflict with the general stasis of the complex compared with taxa outside the complex. However, the ability of *Tetramorium* gynes to fly might weaken this argumentation of isolation. Second, if temperature was the driving factor, we would expect an increase of crypsis not only with altitude but also with latitude, because temperature but not ruggedness is latitude-correlated. However, our results suggest the opposite: Crypsis decreases with latitude; thus, our results point rather at ruggedness than temperature as factor driving the increase of crypsis with altitude. If the weak positive relation between p-values of correct species identification and latitude is not a random effect, we explain it by low p-values of samples collected in Mediterranean mountainous landscapes, which are isolated from northern populations in, for example, *Tetramorium alpestre*, *T*. *caespitum*, and *T*. *impurum*. In any case, the decrease of crypsis with latitude is in line with other studies that revealed high cryptic diversity in the tropics^76–78^.

We wonder if the relation between crypsis and ruggedness is limited to the *T*. *caespitum* complex or if this pattern holds more broadly. Some biodiversity researchers emphasize the high frequency of ‘cryptic species’ in high-mountain systems^79–84^; however, they did not include morphometric data, and thus they did not demonstrate quantitatively that crypsis increases with ruggedness. To summarize, based on own results and literature records, crypsis seems to increase with increasing terrain ruggedness and not with decreasing temperature.

## Conclusion

Based on nuDNA data, the *Tetramorium caespitum* complex is a monophylum with respect to the selected set of *T*.*-caespitum*-group species outside the complex. This finding is in line with the morphology of workers, gynes, and males. However, data of social parasites using species of the *T*. *caespitum* complex as host are lacking in our study. We detected a slow loss of morphological similarity in workers over evolutionary time. Thus, stasis and not convergence likely is the evolutionary force behind morphological crypsis in this ant species complex. Since the distance of only one morphological character correlated positively with ecological distance, we consider concerted evolution between morphological traits and ecology as an exception. The *T*. *caespitum* complex worker body seems to fit in various environments without species-specific morphological adaptations. Nests of species occurring in higher altitudes have smaller p-values of correct species identification and are thus more cryptic. This pattern possibly results from intraspecific variability due to isolation of mountainous populations.

The number of eleven cryptic species makes the *Tetramorium caespitum* complex an ideal study system to test general questions of the evolution of morphological crypsis. However, at least two further interesting topics remain for future research. First, the lack of a relation between morphology and ecological niches calls for searching species-specific adaptations outside of worker morphology. Hence, searching for physiological or behavioural adaptations of cryptic species living in different environments seems worthwhile. Second, the relation of terrain ruggedness and crypsis requires in-depth investigations also in other taxa^2^. A greater fraction of cryptic species in higher altitude would be relevant for conservation biology: The elevational shrinking of surface area^85^ increases the threat by climate warming with increasing altitude^86^ – if many species in higher altitudes actually comprise up to now unknown cryptic species, the true number of affected species will be higher than currently expected.

## Electronic supplementary material

Supplementary Material
