# Supplementary material for: Evolution of morphological crypsis in the Tetramorium caespitum ant species complex (Hymenoptera: Formicidae)
Source: Sci Rep. 2018 Aug 22;8:12547. doi: 10.1038/s41598-018-30890-z (PMC6105586; doi:10.1038/s41598-018-30890-z)
Supplement: Supplementary file 1 — Supplementary Material [file 41598_2018_30890_MOESM1_ESM.pdf]

Evolution of morphological crypsis in the *Tetramorium caespitum* ant species complex (Hymenoptera: Formicidae)

Herbert C. Wagner, Alexander Gamisch, Wolfgang Arthofer, Karl Moder, Florian M. Steiner & Birgit C. Schlick-Steiner

Supplementary Table S1. Complete names of sequenced genes, number of sequenced basepairs (bp), and GenBank accession numbers of the sequences produced in this study.

Gene	size (bp)	GenBank accession
Elongation Factor 1 alpha F1	1443	MH281550–MH281564
Elongation Factor 1 alpha F2	913	MH281475–MH281489
Arginine kinase	1167	MH281535–MH281549
Triose-phosphate isomerase	2840	MH281490–MH281504
Wingless	2096	MH281505–MH281519
Long-wavelength rhodopsin	431	MH293554–MH293568
Abdominal-A	1768	MH281520–MH281534

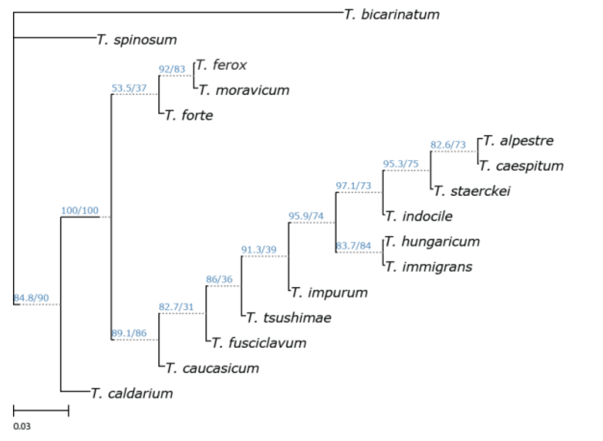

Supplementary Figure S1. Maximum likelihood tree of all *Tetramorium* species included in this study except *T. semilaeve* and using *T. spinosum* as additional outgroup (for details, see text) based on seven nuclear genes (Tab. S1). At nodes, the results of the Shimodaira-Hasegawa branch length tests (before slash) and bootstrapping (after slash) are given.

Abbreviations and definitions of the worker traditional morphometrics characters

Supplementary Table S2. Abbreviations and definitions of the worker traditional morphometrics characters. For illustrations, see Wagner *et al.*[1].

Acronym	Definition
CL	Maximum cephalic length in median line; head is carefully tilted to position with true maximum; excavations of occiput and / or clypeus reduce CL. Peaks due to sculpture are ignored and only valleys are considered.
CS	Arithmetic mean of CL and CW.
CW	Maximum cephalic width across eyes.
dAN	Minimum distance between antennal fossae; measured in dorsofrontal view.
EL	Maximum diameter of one eye. All structurally defined ommatidia, pigmented or not, are included.
EW	Minimum diameter of one eye. All structurally defined ommatidia, pigmented or not, are included.
FL	Maximum distance between external margins of frontal lobes. If this distance is not

	defined because frontal carinae constantly converge frontad, FL is measured at FRS level (definition of FRS see Seifert 2003[2]) as distance between the outer margins of frontal carinae.
HFL	Length of hind femur in dorsal view. Second trochanter, which could appear to be portion of femur, must not be mistakenly included.
MC1TG	Quantification of stickman-like or reticulate microsculpture units on base of 1st gastral tergite (use > 150 × magnification): Number of connected lines building units and being separated by line intersections and by flections angled > 10° is counted. Also very short lines are full counts. Arithmetic means of at least three units per worker are taken.
ML	Mesosoma length measured in dorsal view from caudalmost portion of propodeum to dorsofrontal corner of pronotal slope (i.e., where coarsely structured dorsal shield meets finely structured pronotal neck); equivalent measuring also possible in lateral view.
MPPL	Distance between most anteroventral point of metapleuron and most dorsocaudal point of propodeal lobe in lateral view. If there are two points coming into question to be dorsocaudalmost point on propodeal lobe, the one which is provided with a carina is taken.
MPSP	Distance between most anterioventral point of lateral metapleuron and distalmost point of propodeal spine (it does not need to be uppermost point of spine).
MPST	Distance between most anterioventral point of metapleuron and center of propodeal stigma.
MtpW	Maximum metapleuron width measured in dorsal view. (In most cases, maximum is at caudal and in few cases at central or frontal region of metapleuron.)
MW	Maximum mesosoma width.
PEH	Petiole height. Measured from uppermost point of concave ventral margin to node top.
PEL	Petiole length. Measured in lateral view from center of petiolar stigma to caudal margin of petiole (both measuring points on same focal level).
PEW	Maximum petiole width.
PLSP	Distance between most dorsocaudal point of propodeal lobe (if there are two points coming into question to be dorsocaudalmost point of propodeal lobe, the one which is provided with a carina is taken) and distalmost point of propodeal spine (it does not need to be uppermost point of spine).
PLST	Distance between most dorsocaudal point of propodeal lobe and center of propodeal stigma.

PnHL	Length of hair at frontolateral corner of pronotum. Take longer hair of both sides.
PoOc	Postocular distance. Using cross-scaled ocular micrometer, head is adjusted to measuring position of CL; caudal measuring point: median posterior margin of head, microsculpture peaks are ignored and valleys are considered; frontal measuring point: median head crossing line between posterior eye margins.
POTCos	Number of postoculo-temporal costae and costulae. With head in lateral view and longitudinal axis of head adjusted horizontally, counted by focussing along perpendicular line from caudalmost point of eye down to underside of head. Costae / costulae just touching measuring line are counted as 0.5, those positioned just at ventral margin of head silhouette are not counted. Arithmetic mean of both sides.
PPH	Maximum postpetiole height.
PPL	Postpetiole length; distance from center of postpetiolar stigma to caudalmost intersection point of tergite and sternite (both measuring points at same focal level).
Ppss	Maximum height of smooth and shiny area on lateral side of propodeum. This area is brought into visual plane; a line is positioned perpendicular to main costae on propodeum and maximum height of smooth and shiny area without any costulae and costae is measured. Take arithmetic means of both sides.
PPW	Maximum postpetiole width.
PreOc	Preocular distance in lateral view. Measured as minimum distance between anterior eye margin and sharp frontal margin of gena.
RTI	Distance between tops of ridges between antennal fossae and clypeus. Tops are defined as dorsofrontalmost points of ridges, provided with a costa on clypeus. Measured in dorsoanterior view.
SLd	Maximum scape length, including scape lobe, excluding articular condyle.
SPST	Distance between distalmost point of propodeal spine (it does not need to be uppermost point of spine) and center of propodeal stigma.
SPWI	Maximum distance between outer margins of propodeal spines. Measured in dorsoanterior view.

References

1. Wagner, H. C. *et al.* Light at the end of the tunnel: integrative taxonomy delimits cryptic species in the *Tetramorium caespitum* complex (Hymenoptera: Formicidae). *Myrmecol. News* **25,** 95–130 (2017).

2. Seifert, B. The ant genus *Cardiocondyla* (Insecta: Hymenoptera: Formicidae) – a taxonomic revision of the *C. elegans*, *C. bulgarica*, *C. batesii*, *C. nuda*, *C. shuckardi*, *C. stambuloffii*, *C. wroughtonii*, *C. emeryi*, and *C. minutior* species groups. *Ann. Naturhistorischen Mus. Wien Ser. B* **104,** 203–338 (2003).
